# Cardiovascular Outcomes Associated with Romosozumab Versus Denosumab in Chronic Kidney Disease

**DOI:** 10.3390/medicina62071302

**Published:** 2026-07-06

**Authors:** Jheng-Yan Chen, Tse-Yu Chen, Kuan-Kai Tung, Ya-Lien Deng, Cheng-Ying Lee, Chi-Ruei Li, Hsu-Tung Lee

**Affiliations:** 1 Department of Neurosurgery, Neurological Institute, Taichung Veterans General Hospital, No. 1650, Taiwan Boulevard Sect. 4, Taichung 40705, Taiwan; abctom789@yahoo.com.tw (J.-Y.C.); terry78423@gmail.com (T.-Y.C.); cklove5212@gmail.com (C.-Y.L.); leesd2001@hotmail.com (H.-T.L.); 2 Doctoral Program in Translational Medicine, National Chung Hsing University, Taichung 40227, Taiwan; 3 Department of Orthopedics, Taichung Veterans General Hospital, Taichung 40705, Taiwan; david02200918@gmail.com; 4 Department of Nursing, Taichung Veterans General Hospital, Taichung 40705, Taiwan; cfalsm@vghtc.gov.tw; 5 Department of Neurosurgery, Taichung Veterans General Hospital Puli Branch, Nantou County 545402, Taiwan; 6 Graduate Institute of Clinical Medicine, Chung Shan Medical University, Taichung 40201, Taiwan; 7 Department of Post-Baccalaureate Medicine, College of Medicine, National Chung Hsing University, Taichung 40227, Taiwan

**Keywords:** cardiovascular outcomes, chronic kidney disease, denosumab, osteoporosis, romosozumab

## Abstract

*Background and Objective:* Romosozumab carries a warning for potential severe cardiovascular events, while denosumab is widely used for osteoporosis but requires safety considerations in advanced chronic kidney disease (CKD). Given the limited direct real-world evidence comparing these treatments, in this study, we aimed to compare the cardiovascular and survival outcomes associated with romosozumab versus denosumab in adults with CKD. *Materials and Methods:* In this retrospective propensity score-matched cohort study, we utilized de-identified electronic health records from the TriNetX Global Collaborative Network, where eligible participants were adults aged 40 to 90 years with CKD who initiated either romosozumab or denosumab. Patients with bone/bone marrow malignancies or recent acute cardiovascular events were excluded. Following 1:1 propensity score matching based on demographics, diagnoses, medications, and laboratory characteristics, patients were followed for up to 1095 days. The primary outcome was a composite cardiovascular measure (all-cause mortality, acute myocardial infarction, or cerebrovascular event), while secondary outcomes included the individual components of the composite outcome and acute heart failure. Outcomes were evaluated using fixed-window cumulative risks, risk ratios (RRs), odds ratios, and hazard-ratio estimates. *Results:* After 1:1 propensity score matching, 1201 patients remained in each cohort; the mean age was 74.1 years in the romosozumab cohort and 74.2 years in the denosumab cohort, and 94.9% and 93.8%, respectively, were women. Romosozumab was associated with lower 1095-day cumulative risk of the composite cardiovascular outcome than denosumab (12.6% vs. 18.8%; RR, 0.668 [95% CI, 0.553–0.808]), as well as lower cumulative risk of cerebrovascular event (5.0% vs. 7.0%; RR, 0.714 [95% CI, 0.518–0.985]), all-cause mortality (6.6% vs. 9.5%; RR, 0.693 [95% CI, 0.526–0.913]), acute myocardial infarction (3.8% vs. 6.2%; RR, 0.613 [95% CI, 0.429–0.878]), and heart failure (2.7% vs. 6.1%; RR, 0.438 [95% CI, 0.292–0.659]). *Conclusions:* In this propensity score-matched EHR cohort of adults with CKD, cardiovascular and survival estimates associated with romosozumab versus denosumab varied by follow-up window and analytic approach. Although 1095-day fixed-window cumulative risks were lower in the romosozumab cohort, corresponding time-to-event estimates were neutral or directionally inconsistent. These findings should not be interpreted as evidence of cardioprotection or causal superiority but rather as showing no clear and consistent excess cardiovascular risk signal for romosozumab compared with denosumab.

## 1. Introduction

As a critical public health challenge worldwide, osteoporosis is a primary contributor to fragility fractures, physical impairment, loss of autonomy, and premature death in the older adult population [1,2,3]. The treatment of osteoporosis in patients with CKD is clinically challenging because skeletal fragility, altered mineral metabolism, and cardiovascular disease often coexist [4,5,6,7]. Disruptions in bone turnover and mineral homeostasis are driven by CKD-mineral and bone disorder; consequently, advanced kidney disease carries a dangerously high baseline risk for severe outcomes, including vascular calcification, heart attack, stroke, and death [6,7,8,9].

As a monoclonal antibody against sclerostin, romosozumab lowers osteoporotic fracture risk in highly susceptible patients by boosting bone formation and decreasing bone resorption, which leads to rapid bone density gains [10,11,12,13,14]. Nevertheless, cardiovascular safety continues to be a primary clinical issue. While the FRAME trial [15] demonstrated no significant disparity in cardiovascular outcomes, the ARCH trial [12] observed a higher incidence of severe cardiovascular events during the 12-month romosozumab period relative to alendronate. Subsequent randomized-trial syntheses and real-world studies have not consistently confirmed an excess cardiovascular risk, but uncertainty remains, particularly in patients with high baseline cardiovascular risk [16,17,18,19].

Denosumab is a receptor activator of nuclear factor kappa-B ligand (RANKL) inhibitor administered biannually. It serves as a therapeutic option for the management of osteoporosis and has also been utilized in patients with impaired kidney function [20,21]. Nevertheless, denosumab can cause severe hypocalcemia in advanced CKD, especially among patients with CKD-mineral and bone disorder, and current labeling includes a boxed warning for severe hypocalcemia in this population [22]. The cardiovascular safety of denosumab has generally appeared neutral in placebo-controlled osteoporosis trials, but comparative evidence versus active treatments and data in advanced kidney disease remain mixed [23,24,25,26].

Direct evidence comparing romosozumab with denosumab in CKD is limited. This question is clinically relevant because both agents are used in patients at high fracture risk, yet CKD patients often have substantial baseline cardiovascular vulnerability and may experience altered bone–vascular signaling. Accordingly, this study compared cardiovascular and survival outcomes associated with romosozumab versus denosumab among adults with CKD using a large federated electronic health record network. We hypothesized that romosozumab would not be associated with higher cardiovascular risk than denosumab in patients with CKD.

## 2. Materials and Methods

### 2.1. Data Sources

This retrospective propensity score-matched cohort study analyzed de-identified EHR data from 170 healthcare organizations within the TriNetX Global Collaborative Network (as of 16 March 2026). The extracted dataset, whose quality has been previously validated, included patient demographics, diagnoses, procedures, medications, lab results, and mortality data [27,28].

### 2.2. Study Design and Cohort Selection

Adults aged 40 to 90 years with CKD (International Classification of Diseases, Tenth Revision, Clinical Modification [ICD-10-CM] code N18) documented before study-drug initiation were eligible, and the index date was the first qualifying record of the study drug. The romosozumab cohort included patients with a record of romosozumab (RxNorm 2123126) or injection of romosozumab-aqqg (HCPCS J3111), while the denosumab cohort included patients with a record of denosumab (RxNorm 993449), injection of denosumab (HCPCS J0897), or denosumab-bbdz biosimilar injection (HCPCS Q5136).

Patients with primary or secondary malignant neoplasm of bone or bone marrow before treatment initiation were excluded. Both cohorts excluded patients with acute cardiovascular events during the year before the index date, including cardiac arrest, acute myocardial infarction, other acute ischemic heart disease, transient ischemic attack, cerebrovascular syndromes, nontraumatic intracerebral or intracranial hemorrhage, cerebral infarction, and acute systolic or diastolic heart failure. To reduce treatment crossover, romosozumab users were required to have no denosumab exposure during the 6 months before and 3 years after the index date, and denosumab users were required to have no romosozumab exposure during the corresponding period. Both cohorts were required to have at least 2 records of the assigned study drug; repeat records were required within 3 months for romosozumab and within 225 days for denosumab, consistent with expected dosing intervals. These post-index exposure requirements were part of the source cohort definition and are addressed as a limitation.

Given the standard 12-month treatment duration of romosozumab and the observed discordance between fixed-window cumulative risks and time-to-event estimates, we performed an additional 365-day treatment-period analysis. This analysis used the same cohort definitions, exclusion criteria, outcome definitions, and propensity score-matching covariates as the extended 1095-day analysis, but restricted the outcome window to day 1 through day 365 after the index date. Propensity score matching was repeated for this analysis, resulting in a separately matched cohort. Fixed-window cumulative risks, risk differences, risk ratios, odds ratios, and hazard ratios for the 365-day window were generated by the TriNetX platform. Because this analysis was based on a separately matched cohort and the database did not provide patient-level treatment-adherence or dosing data, it was interpreted as an additional clinically aligned treatment-period analysis rather than a causal on-treatment estimate.

Because the second study-drug record occurred after the index date, the main persistence-restricted design may have introduced an immortal-time structure: patients had to remain alive, under observation, and eligible for cohort qualification until the repeat-dose window was satisfied. Therefore, we performed an additional ITT-like initiator sensitivity analysis to assess the potential influence of post-index exposure requirements and immortal-time bias. In this analysis, patients were assigned to the romosozumab or denosumab cohort according to their first recorded study-drug exposure, without requiring a second post-index drug record or repeat dosing within prespecified windows. Patients were followed from day 1 through day 1095 after treatment initiation according to their initial treatment group. Subsequent treatment switching or discontinuation was not used as an exclusion criterion in this ITT-like analysis.

To evaluate whether early event timing and post-index repeat-dose qualification requirements influenced the discordance between fixed-window cumulative risks and time-to-event estimates, we performed an additional day-225 landmark/delayed-start sensitivity analysis. This analysis used the same cohort definitions, exclusion criteria, outcome definitions, and propensity score-matching covariates as the extended 1095-day analysis, but the outcome window was shifted to start 226 days after the index date and end 1095 days after the index date. The 225-day landmark was selected because it corresponded to the maximum repeat-dose qualification window used for denosumab in the original cohort definition. Propensity score matching was repeated for this analysis. Fixed-window cumulative risks, risk differences, risk ratios, odds ratios, and hazard ratios were generated by the TriNetX platform. Because patients with outcomes before the delayed-start window were not excluded by the platform setting, this analysis was interpreted as a delayed-start late-window sensitivity analysis rather than a strict event-free landmark analysis.

Finally, an eGFR-restricted sensitivity analysis was performed to evaluate patients with reduced kidney function within the diagnosis-defined CKD cohort. This analysis retained the original CKD diagnosis requirement, defined as ICD-10-CM N18 documented before treatment initiation, and further restricted eligibility to patients with a recorded pre-index eGFR ≤ 60 mL/min/1.73 m^2^. The same exclusion criteria, propensity score-matching approach, outcome definitions, and 1095-day follow-up window were applied. Because the TriNetX query identified recorded pre-index eGFR values at or below 60 mL/min/1.73 m^2^ rather than confirming CKD chronicity or adjudicating CKD stage using repeated longitudinal eGFR measurements, this analysis was interpreted as a reduced-kidney-function sensitivity analysis.

Laboratory covariates were included when recorded values were available in the TriNetX baseline-characteristics output. Missing laboratory values were not used as exclusion criteria, and no complete-case restriction or investigator-initiated imputation was performed. Patients with missing values for individual laboratory variables remained eligible for propensity score matching. For each laboratory variable, summary statistics were calculated among patients with available values, and the number and proportion of patients with available data were reported textually in the Results section. Because patient-level laboratory data were not available, additional multiple imputation or missing-data modeling could not be performed.

### 2.3. Baseline Covariates and Propensity Score Matching

Propensity score matching was performed using the TriNetX platform on all listed baseline characteristics in the data report. Covariates included demographic characteristics, cardiovascular and metabolic comorbidities, chronic obstructive pulmonary disease, medication classes relevant to cardiovascular risk and osteoporosis care, and laboratory values including body mass index, blood pressure, serum creatinine, glucose, hemoglobin A1c, hemoglobin, lipid values, estimated glomerular filtration rate (eGFR), and serum calcium. Patients were matched 1:1 using the TriNetX propensity score algorithm. Covariate balance was assessed using standardized mean differences (SMDs), with absolute SMD values below 0.10 considered acceptable.

To improve readability, the main manuscript presents selected clinically relevant baseline characteristics after propensity score matching in Table 1. The complete covariate balance, including all variables used in propensity score matching and their SMDs, is provided in Supplementary Table S1.

### 2.4. Outcomes

The primary outcome was a composite cardiovascular outcome defined as all-cause mortality, acute myocardial infarction, or cerebrovascular event. Cerebrovascular events included transient ischemic attack, vascular syndromes of the brain in cerebrovascular diseases, nontraumatic intracerebral hemorrhage, other nontraumatic intracranial hemorrhage, and cerebral infarction. Acute myocardial infarction included cardiac arrest, acute myocardial infarction, and other acute ischemic heart diseases. Secondary outcomes were the individual components of the composite outcome and acute heart failure, defined as acute systolic, acute-on-chronic systolic, acute diastolic, or acute-on-chronic diastolic heart failure. The primary follow-up window extended from day 1 through day 1095 after the index date. This window was selected to evaluate longer-term, real-world cardiovascular and survival outcomes after treatment initiation. Because romosozumab is generally administered for a finite 12-month course, whereas denosumab is administered every 6 months and may be continued longer term, the 1095-day analysis should not be interpreted as a comparison of equivalent continuous treatment exposure. In the original cohort definition, comparator crossover was restricted to reduce treatment contamination; specifically, denosumab exposure was excluded in the romosozumab cohort during follow-up, and romosozumab exposure was excluded in the denosumab cohort. Subsequent non-comparator antiresorptive therapy after romosozumab completion, including bisphosphonate therapy, could not be reliably reconstructed from the aggregated TriNetX output.

### 2.5. Statistical Analysis

Fixed-window cumulative risks were calculated as the proportion of patients in each matched cohort with the outcome during days 1 through 1095. Risk differences, RRs, odds ratios, and 95% CIs were generated using the TriNetX platform, and hazard ratios were obtained as complementary time-to-event estimates, while proportionality testing was reported using the TriNetX platform. Because the source data included post-index exposure requirements and did not provide patient-level time-to-event data for independent modeling, analyses were interpreted as comparative real-world associations rather than causal treatment effects.

### 2.6. Ethics Statement

TriNetX data are de-identified in accordance with the Health Insurance Portability and Accountability Act, and informed consent was therefore waived. The study protocol was reviewed by the Institutional Review Board of Taichung Veterans General Hospital (approval no.: CE25926B). This study followed the Strengthening the Reporting of Observational Studies in Epidemiology (STROBE) reporting guideline [29].

## 3. Results

### 3.1. Study Population and Baseline Characteristics

Before matching, 1201 patients in the romosozumab cohort and 21,772 patients in the denosumab cohort were available for propensity score matching; after 1:1 matching, 1201 patients remained in each cohort. Before matching, romosozumab users were younger on average than denosumab users and were more often women. Several cardiovascular, metabolic, medication, and kidney-function characteristics also differed before matching, as shown in the complete covariate-balance table in Supplementary Table S1.

After matching, demographic and clinical variables were generally balanced. The mean age was 74.1 years in the romosozumab cohort and 74.2 years in the denosumab cohort, and 94.9% and 93.8%, respectively, were women. The prevalence of hypertension was 70.0% vs. 68.3%, ischemic heart disease was 18.2% vs. 18.6%, atrial fibrillation or flutter was 12.4% vs. 12.4%, heart failure was 14.3% vs. 14.6%, and diabetes mellitus was 31.3% vs. 30.5%; the mean eGFR was 52.6 vs. 51.7 mL/min/1.73 m^2^. Most post-matching SMDs were below 0.10. Residual imbalance persisted for selected laboratory variables, including hemoglobin A1c, HDL cholesterol, and serum calcium, as detailed in Supplementary Table S1.

Laboratory covariate availability varied across variables in the matched cohorts. Kidney-function and mineral-metabolism variables were relatively well captured: eGFR values were available for 1086/1201 (90.4%) romosozumab users and 1072/1201 (89.3%) denosumab users, serum creatinine for 1075/1201 (89.5%) and 1063/1201 (88.5%), and serum calcium for 971/1201 (80.8%) and 951/1201 (79.2%), respectively. In contrast, several metabolic and lipid variables had substantial missingness: hemoglobin A1c was available for 580/1201 (48.3%) and 577/1201 (48.0%), HDL cholesterol for 583/1201 (48.5%) and 567/1201 (47.2%), and LDL cholesterol for 621/1201 (51.7%) and 611/1201 (50.9%), respectively. Laboratory summary statistics were calculated among patients with available recorded values only; missing laboratory values were not imputed and were not used as exclusion criteria.

### 3.2. Cardiovascular and Survival Outcomes Through 1095 Days

In the propensity score-matched cohorts, romosozumab was associated with a lower fixed-window cumulative risk of the composite cardiovascular outcome than denosumab (151 of 1201 [12.6%] vs. 226 of 1201 [18.8%]; risk difference, −0.062 [95% CI, −0.091 to −0.033]; RR, 0.668 [95% CI, 0.553–0.808]; odds ratio, 0.620 [95% CI, 0.496–0.776]).

Romosozumab was also associated with lower cumulative risks of each secondary outcome. The risk of cerebrovascular event was 5.0% with romosozumab vs. 7.0% with denosumab (RR, 0.714 [95% CI, 0.518–0.985]), all-cause mortality was 6.6% vs. 9.5% (RR, 0.693 [95% CI, 0.526–0.913]), acute myocardial infarction was 3.8% vs. 6.2% (RR, 0.613 [95% CI, 0.429–0.878]), and acute heart failure was 2.7% vs. 6.1% (RR, 0.438 [95% CI, 0.292–0.659]) (Table 2).

The corresponding hazard-ratio estimates were 1.181 (95% CI, 0.958–1.455) for the composite outcome, 1.241 (95% CI, 0.886–1.738) for cerebrovascular event, 1.306 (95% CI, 0.976–1.747) for all-cause mortality, 1.096 (95% CI, 0.755–1.590) for acute myocardial infarction, and 0.720 (95% CI, 0.472–1.097) for heart failure. Thus, fixed-window cumulative risk analyses favored romosozumab, whereas time-to-event estimates were attenuated or directionally inconsistent and did not exclude the null.

### 3.3. Cardiovascular and Survival Outcomes Through 365 Days

During the 365-day treatment-period analysis, fixed-window cumulative risks were broadly similar between the romosozumab and denosumab cohorts. The risk of the composite cardiovascular outcome was 8.3% in the romosozumab cohort and 7.9% in the denosumab cohort (RR, 1.049 [95% CI, 0.785–1.402]). The corresponding risks were 3.3% vs. 3.1% for cerebrovascular events (RR, 1.063 [95% CI, 0.661–1.708]), 4.2% vs. 3.4% for all-cause mortality (RR, 1.229 [95% CI, 0.793–1.903]), 2.6% vs. 2.6% for acute myocardial infarction (RR, 1.000 [95% CI, 0.591–1.693]), and 1.7% vs. 2.6% for acute heart failure (RR, 0.667 [95% CI, 0.369–1.203]).

In time-to-event analyses, the HRs were 1.425 (95% CI, 1.051–1.931) for the composite cardiovascular outcome, 1.405 (95% CI, 0.865–2.282) for cerebrovascular events, 1.741 (95% CI, 1.113–2.725) for all-cause mortality, 1.326 (95% CI, 0.776–2.264) for acute myocardial infarction, and 0.828 (95% CI, 0.455–1.506) for acute heart failure. Kaplan–Meier event-free survival probabilities at 365 days were 88.25% vs. 91.53% for the composite cardiovascular outcome, 95.37% vs. 96.68% for cerebrovascular events, 93.90% vs. 96.33% for all-cause mortality, 96.20% vs. 97.23% for acute myocardial infarction, and 97.68% vs. 97.24% for acute heart failure in the romosozumab and denosumab cohorts, respectively.

### 3.4. ITT-like Initiator Sensitivity Analysis

In the ITT-like initiator sensitivity analysis, which did not require post-index repeat-dose records, 1728 patients were included in each propensity score-matched cohort. Fixed-window cumulative risks remained lower in the romosozumab cohort for the composite cardiovascular outcome (11.3% vs. 21.6%; RR, 0.524 [95% CI, 0.447–0.615]), cerebrovascular events (4.3% vs. 6.9%; RR, 0.617 [95% CI, 0.465–0.818]), all-cause mortality (6.0% vs. 13.4%; RR, 0.444 [95% CI, 0.355–0.554]), acute myocardial infarction (3.6% vs. 6.0%; RR, 0.606 [95% CI, 0.446–0.822]), and acute heart failure (2.7% vs. 4.6%; RR, 0.588 [95% CI, 0.412–0.837]).

In contrast to the original persistence-restricted analysis, the time-to-event estimates were directionally concordant with the fixed-window risk estimates. The HRs were 0.809 (95% CI, 0.679–0.964) for the composite cardiovascular outcome, 0.969 (95% CI, 0.722–1.299) for cerebrovascular events, 0.731 (95% CI, 0.578–0.925) for all-cause mortality, 0.926 (95% CI, 0.675–1.271) for acute myocardial infarction, and 0.905 (95% CI, 0.629–1.304) for acute heart failure.

### 3.5. Day-225 Landmark Analysis

In the day-225 landmark/delayed-start sensitivity analysis, fixed-window cumulative risks from day 226 through day 1095 were lower in the romosozumab cohort than in the denosumab cohort across all evaluated outcomes. The risk of the composite cardiovascular outcome was 8.7% in the romosozumab cohort and 17.8% in the denosumab cohort (RR, 0.489 [95% CI, 0.396–0.604]). The corresponding risks were 3.3% vs. 6.8% for cerebrovascular events (RR, 0.489 [95% CI, 0.342–0.698]), 4.9% vs. 10.2% for all-cause mortality (RR, 0.485 [95% CI, 0.363–0.647]), 2.1% vs. 5.1% for acute myocardial infarction (RR, 0.409 [95% CI, 0.263–0.636]), and 1.6% vs. 4.8% for acute heart failure (RR, 0.339 [95% CI, 0.208–0.552]).

In contrast, the corresponding time-to-event estimates were attenuated and did not demonstrate statistically significant differences. The HRs were 1.043 (95% CI, 0.831–1.309) for the composite cardiovascular outcome, 1.020 (95% CI, 0.706–1.473) for cerebrovascular events, 1.071 (95% CI, 0.792–1.447) for all-cause mortality, 0.917 (95% CI, 0.583–1.440) for acute myocardial infarction, and 0.731 (95% CI, 0.444–1.203) for acute heart failure. Kaplan–Meier event-free survival probabilities at the end of the delayed-start window were 76.68% vs. 75.29% for the composite cardiovascular outcome, 89.53% vs. 90.57% for cerebrovascular events, 87.58% vs. 85.44% for all-cause mortality, 94.01% vs. 92.30% for acute myocardial infarction, and 95.04% vs. 93.00% for acute heart failure in the romosozumab and denosumab cohorts, respectively. These findings indicate that lower fixed-window late cumulative risks were not consistently supported by time-to-event estimates.

### 3.6. eGFR-Restricted Sensitivity Analysis

To further evaluate patients with reduced kidney function, we performed an additional analysis restricted to diagnosis-defined CKD patients with a recorded pre-index eGFR ≤ 60 mL/min/1.73 m^2^. After propensity score matching, fixed-window cumulative risks were lower in the romosozumab cohort for the composite cardiovascular outcome (13.6% vs. 20.7%; RR, 0.657 [95% CI, 0.529–0.816]), all-cause mortality (6.9% vs. 11.4%; RR, 0.600 [95% CI, 0.438–0.821]), acute myocardial infarction (4.0% vs. 6.5%; RR, 0.611 [95% CI, 0.401–0.932]), and acute heart failure (2.2% vs. 7.7%; RR, 0.281 [95% CI, 0.168–0.470]). The cumulative risk of cerebrovascular events was numerically lower but not statistically significant (5.4% vs. 6.9%; RR, 0.789 [95% CI, 0.541–1.153]).

In time-to-event analyses, the HRs were 1.208 (95% CI, 0.949–1.538) for the composite cardiovascular outcome, 1.406 (95% CI, 0.944–2.093) for cerebrovascular events, 1.190 (95% CI, 0.853–1.662) for all-cause mortality, 1.118 (95% CI, 0.720–1.736) for acute myocardial infarction, and 0.483 (95% CI, 0.285–0.819) for acute heart failure. Overall, the eGFR-restricted analysis supported lower fixed-window cumulative risks in patients with reduced kidney function, but the time-to-event estimates remained mixed and should be interpreted cautiously.

## 4. Discussion

In this propensity score-matched cohort of adults with CKD, the interpretation of cardiovascular outcomes differed according to the analytic time window and estimands. In the extended 1095-day analysis, fixed-window cumulative risks were lower in the romosozumab cohort than in the denosumab cohort for the composite cardiovascular outcome, cerebrovascular events, all-cause mortality, acute myocardial infarction, and acute heart failure. However, the corresponding time-to-event estimates were neutral or directionally inconsistent. In the additional 365-day treatment-period analysis, fixed-window risks were generally similar between groups, whereas hazard ratios for the composite cardiovascular outcome and all-cause mortality were higher in the romosozumab cohort. Taken together, these findings do not support a cardioprotective interpretation of romosozumab. Rather, they suggest that romosozumab was not consistently associated with lower cardiovascular risk than denosumab and that estimates must be interpreted cautiously in light of potential informative censoring, post-index exposure requirements, and residual confounding.

The findings should be interpreted in the context of the ongoing debate regarding romosozumab cardiovascular safety. Romosozumab demonstrated strong antifracture efficacy in pivotal randomized trials, but the ARCH trial reported more adjudicated cardiovascular serious adverse events with romosozumab than with alendronate during the first 12 months, whereas FRAME did not show a comparable imbalance [12,15]. Current labeling continues to warn that romosozumab may increase the risk of myocardial infarction, stroke, and cardiovascular death [11]. More recent meta-analyses and observational studies have generally been less definitive, suggesting that the cardiovascular signal may depend on patient selection, comparator choice, baseline cardiovascular burden, and analytic approach [17,18,19,23,24,30]. The present CKD-focused comparison against denosumab did not identify higher cumulative cardiovascular risk with romosozumab, but the discordance between cumulative-risk and survival estimates precludes strong causal inference.

The lower fixed-window cumulative risks observed with romosozumab should be interpreted cautiously because they were not corroborated by the corresponding time-to-event analyses. Although the risk analyses suggested fewer cardiovascular and mortality events in the romosozumab group, the results did not demonstrate a consistent or statistically significant time-to-event advantage over denosumab. Several methodological factors may explain this discordance. The cohort definitions required repeated post-index study-drug records and absence of crossover to the comparator during follow-up, which may have selected patients who remained alive, clinically stable, and under observation long enough to continue therapy. In addition, survival analyses censored patients after the last recorded clinical fact; therefore, differential follow-up intensity or informative censoring may have influenced the relationship between fixed-window cumulative-risk estimates and time-to-event estimates.

The discordance between fixed-window cumulative risks and time-to-event estimates is a key interpretive issue in this study. Fixed-window cumulative risks estimate the proportion of patients with an event within a prespecified period, but they do not incorporate event timing and may be influenced by differential follow-up intensity, incomplete event capture, and post-index cohort qualification requirements. In contrast, hazard ratios incorporate the timing of events, but their validity depends on assumptions such as proportional hazards and non-informative censoring, which may not be fully satisfied in federated EHR data. In the present study, the lower 1095-day cumulative risks observed with romosozumab were not consistently supported by the corresponding hazard-ratio estimates. Therefore, we did not consider the fixed-window RRs to provide stronger causal evidence than the HRs. Rather, the discordance across estimands led us to interpret the results conservatively as showing no clear and consistent excess cardiovascular risk signal for romosozumab compared with denosumab, without evidence of cardiovascular protection or causal superiority.

Residual confounding remains possible despite propensity score matching. Although most measured covariates achieved acceptable post-matching balance, residual imbalance persisted for hemoglobin A1c, HDL cholesterol, and serum calcium. These variables are clinically relevant in CKD because they may reflect metabolic risk, nutritional status, mineral metabolism, and CKD–mineral and bone disorder severity, all of which may influence cardiovascular outcomes and treatment selection. In addition, unmeasured factors such as osteoporosis severity, frailty, fracture history, dialysis status, CKD–mineral and bone disorder severity, calcium or vitamin D supplementation, prior osteoporosis treatment, and clinician selection of therapy may have influenced treatment assignment and outcomes. Because the TriNetX output was aggregated and patient-level data were unavailable, investigator-specified doubly robust adjustment or multiple imputation could not be performed. Therefore, the findings should be interpreted as observational associations rather than evidence of cardiovascular protection or superiority.

Treatment sequencing after romosozumab completion is another important consideration when interpreting the extended 1095-day results. Romosozumab is generally administered as a finite 12-month course, after which antiresorptive therapy is commonly used to maintain gains in bone mineral density. Therefore, the objective of the extended follow-up analysis was not to estimate the isolated pharmacologic effect of continuous romosozumab exposure over 3 years but rather to evaluate real-world cardiovascular and survival outcomes after romosozumab initiation as captured in routine practice.

In this context, subsequent antiresorptive therapy after completion of romosozumab was not intentionally prohibited, except for denosumab exposure, which was restricted to reduce comparator crossover and treatment contamination. Thus, subsequent non-denosumab antiresorptive therapy, including bisphosphonates, may have occurred as part of usual post-romosozumab care. However, because the aggregated TriNetX output did not allow reliable reconstruction of post-index treatment sequencing, adherence, discontinuation, or cumulative exposure, the potential influence of bisphosphonate or other sequential therapy on the 1095-day cardiovascular and survival estimates cannot be excluded. For this reason, the 1095-day findings should be interpreted as real-world outcomes after treatment initiation rather than as causal effects attributable solely to romosozumab exposure.

The additional 365-day treatment-period analysis provides important context for interpreting the discordance between the 1095-day fixed-window cumulative-risk estimates and time-to-event estimates. Because romosozumab is generally administered for a 12-month course, the 365-day window is clinically aligned with the expected treatment period. In this analysis, fixed-window cumulative risks were broadly similar between romosozumab and denosumab, rather than consistently favoring romosozumab. However, time-to-event estimates for the composite cardiovascular outcome and all-cause mortality were higher in the romosozumab cohort during the first year. In contrast, the 1095-day fixed-window cumulative risks favored romosozumab, whereas the corresponding hazard ratios were neutral or directionally inconsistent. This pattern suggests that differences in event timing, late event accumulation, differential follow-up intensity, informative censoring, and post-index exposure requirements may have contributed to the discordance between cumulative-risk and time-to-event estimates. Therefore, the lower 1095-day cumulative risks should not be interpreted as evidence of cardiovascular protection.

The 365-day analysis also highlights the challenge of comparing agents with different dosing structures. Romosozumab is administered monthly for up to 12 months, whereas denosumab is typically administered every 6 months. Thus, the first-year comparison reflects two clinically used treatment strategies but not equivalent dosing frequency or cumulative pharmacologic exposure. Moreover, the database did not provide reliable patient-level information on actual administration, adherence, discontinuation, or subsequent antiresorptive therapy after romosozumab completion. These considerations further support a cautious interpretation of the findings as showing no consistent cardiovascular advantage of romosozumab over denosumab, rather than evidence of cardioprotection.

The post-index repeat-dose requirements in the main analysis may have influenced both fixed-window cumulative-risk estimates and hazard-ratio estimates. Because patients were required to have repeat study-drug records within prespecified post-index windows—within 3 months for romosozumab and within 225 days for denosumab—individuals with very early death, cardiovascular events, loss to follow-up, treatment discontinuation, or incomplete medication capture may have been less likely to qualify for the persistence-restricted cohort. This structure may have introduced immortal-time or selection bias and may have contributed to the discordance between the lower fixed-window cumulative risks and the neutral or directionally inconsistent time-to-event estimates. It may also have biased fixed-window cumulative-risk comparisons in favor of romosozumab if early events or early discontinuation were differentially captured or differentially excluded between treatment groups. The ITT-like initiator sensitivity analysis, which removed the post-index repeat-dose requirement, provided insight into the potential influence of this design feature. In that analysis, the RRs and HRs became directionally concordant, suggesting that the original inconsistency may have been influenced by post-index cohort selection, immortal-time structure, or differential censoring. Nevertheless, this sensitivity analysis should not be interpreted as evidence of cardioprotection because treatment switching, adherence, actual injection administration, subsequent antiresorptive therapy, and unmeasured CKD–MBD severity could not be fully captured from the aggregated TriNetX output.

The day-225 landmark/delayed-start analysis provides additional insight into the discordance between fixed-window cumulative-risk estimates and time-to-event estimates. This analysis was designed to examine outcomes after the maximum repeat-dose qualification window used in the original cohort construction, thereby reducing the influence of very early events and early post-index exposure qualification. In this delayed-start window, fixed-window cumulative risks remained lower in the romosozumab cohort for the composite cardiovascular outcome, cerebrovascular events, all-cause mortality, acute myocardial infarction, and acute heart failure. However, the corresponding hazard ratios were neutral, and all confidence intervals crossed 1.0. This pattern suggests that the lower fixed-window cumulative risks should not be interpreted as evidence of a time-to-event cardiovascular advantage or cardioprotection.

Several explanations may account for this persistent discordance. First, the underlying source cohort still incorporated post-index exposure requirements and crossover restrictions, which may have selected patients who survived and remained under observation long enough to satisfy treatment-persistence criteria. Second, the delayed-start analysis did not fully reconstruct a strict event-free landmark cohort because patients with outcomes before the delayed-start window were not excluded by the platform setting. Third, differential follow-up intensity and censoring after the last recorded clinical fact may have affected Kaplan–Meier estimates. Therefore, the landmark/delayed-start findings should be interpreted as a sensitivity analysis supporting cautious interpretation of the main results rather than as definitive evidence of cardiovascular benefit with romosozumab.

The eGFR-restricted sensitivity analysis directly addressed the concern that the diagnosis-defined CKD cohort included patients with eGFR values at or above 60 mL/min/1.73 m^2^. Among patients with documented CKD and recorded pre-index eGFR ≤ 60 mL/min/1.73 m^2^, fixed-window cumulative risks remained lower in the romosozumab cohort for most cardiovascular and survival outcomes. However, time-to-event estimates were again less consistent, with HRs for the composite cardiovascular outcome, cerebrovascular events, all-cause mortality, and acute myocardial infarction crossing 1.0. These findings support the robustness of the cumulative-risk findings among patients with reduced kidney function, but they also reinforce the need for cautious interpretation because lower cumulative risks were not uniformly supported by time-to-event estimates.

This study has several limitations. First, the analysis was observational and based on de-identified electronic health record data, which may be subject to miscoding, incomplete medication capture, and incomplete outcome ascertainment. Exposure was defined using medication and procedure records and therefore may not reflect actual injection administration, adherence, persistence, discontinuation, dose timing, or cumulative dose. This limitation is particularly relevant because romosozumab and denosumab have different dosing structures. Romosozumab is generally administered monthly for a finite 12-month course, whereas denosumab is administered every 6 months and may be continued longer term. Accordingly, the 1095-day outcome window may have captured events occurring after completion of the intended romosozumab course and potentially after subsequent antiresorptive therapy, whereas denosumab exposure may have continued during follow-up. Detailed treatment sequencing after study-drug initiation was not available. In the present cohort definition, denosumab exposure during follow-up was excluded in the romosozumab cohort to reduce comparator crossover; therefore, subsequent antiresorptive therapy, when used, may have included bisphosphonates or other non-denosumab agents. Because post-index bisphosphonate exposure, treatment adherence, discontinuation, dose timing, and cumulative exposure could not be reliably reconstructed from the aggregated TriNetX output, these factors may have influenced the 1095-day cardiovascular and survival estimates.

Second, the post-index exposure requirements in the main analysis, particularly repeated study-drug records and absence of comparator exposure during follow-up, may have introduced selection bias and immortal-time bias. Patients who survived long enough, remained clinically stable, and continued to have records within the health system were more likely to satisfy these criteria. In addition, patient-level time-to-event data were not available for investigator-specified stricter-caliper rematching, doubly robust post-matching adjustment, or independent multivariable modeling. Because TriNetX Kaplan–Meier analyses censor patients after the last recorded clinical fact, differential follow-up intensity and informative censoring may have affected the relationship between fixed-window cumulative-risk estimates and time-to-event estimates.

Third, in the main analysis, CKD status was identified using ICD-10-CM N18 codes documented before treatment initiation. Although baseline kidney-function variables, including eGFR and serum creatinine, were included when available, the main cohort was not adjudicated using laboratory-confirmed CKD chronicity or CKD stage. Therefore, misclassification of CKD status and mixing of CKD stages may have occurred. To partially address this concern, we performed an eGFR-restricted sensitivity analysis among patients with diagnosis-defined CKD and a recorded pre-index eGFR ≤ 60 mL/min/1.73 m^2^. However, the TriNetX query interface did not allow us to operationalize a stricter CKD definition requiring two temporally distinct pre-index eGFR values ≤ 60 mL/min/1.73 m^2^ according to a prespecified chronicity window. Accordingly, the eGFR-restricted analysis should be interpreted as a reduced-kidney-function sensitivity analysis rather than as a definitive laboratory-adjudicated CKD staging analysis. In addition, available laboratory measures did not fully characterize CKD–mineral and bone disorder. Granular data on dialysis status, parathyroid hormone, phosphate, vitamin D status, bone turnover markers, fracture history, calcium or vitamin D supplementation, and CKD-MBD severity were unavailable. These factors are clinically important because they may influence both treatment selection and cardiovascular risk. Therefore, confounding by indication and residual CKD-MBD-related confounding may remain despite propensity score matching.

Fourth, competing risks are particularly relevant in CKD populations because mortality is common and may preclude the observation of nonfatal cardiovascular events. Although all-cause mortality was included as a component of the composite cardiovascular outcome, death may have acted as a competing event for nonfatal myocardial infarction, cerebrovascular events, and heart failure. When competing mortality is substantial, standard Kaplan–Meier methods that treat competing events as censoring may overestimate the cumulative incidence of nonfatal outcomes, and conventional Cox models may not directly reflect the competing-risk cumulative-incidence process. This concern is particularly important if mortality risk, follow-up intensity, or censoring patterns differ between treatment groups. Because patient-level time-to-event data were not available from the aggregated TriNetX output, Fine–Gray subdistribution hazard modeling could not be performed. Therefore, nonfatal cardiovascular outcomes should be interpreted cautiously.

Finally, although the propensity score model included demographic, clinical, medication, and laboratory covariates, residual imbalance persisted for hemoglobin A1c, HDL cholesterol, total cholesterol, and serum calcium. These variables are related to cardiovascular risk and mineral metabolism; therefore, residual confounding cannot be excluded. The findings should also be generalized cautiously because the analysis did not include fracture outcomes, hypocalcemia outcomes, or detailed longitudinal medication exposure after treatment initiation.

## 5. Conclusions

In this propensity score-matched observational EHR cohort of adults with CKD, cardiovascular and survival estimates associated with romosozumab versus denosumab varied by follow-up window and analytic approach. Although 1095-day fixed-window cumulative risks were lower in the romosozumab cohort, these findings were not consistently supported by the corresponding time-to-event estimates, and the 365-day treatment-period analysis did not establish a consistent cardiovascular advantage for romosozumab. Therefore, the present findings should be interpreted as observational associations only and should not be considered evidence that romosozumab reduces cardiovascular risk, provides cardioprotection, or is causally superior to denosumab. At most, the results suggest that romosozumab was not associated with a clear and consistent excess cardiovascular risk signal compared with denosumab in this CKD cohort. Important uncertainty remains because of the finite 12-month romosozumab treatment course, differences in treatment duration and sequencing, potential confounding by indication, residual covariate imbalance, lack of cause-specific mortality data, possible competing-risk and informative-censoring effects, and incomplete information on treatment adherence, subsequent antiresorptive therapy, and CKD–mineral and bone disorder severity. Further patient-level studies with detailed longitudinal medication exposure, CKD-MBD characterization, and adjudicated cardiovascular and cause-specific mortality outcomes are needed.

## Figures and Tables

**Table 1:** Selected baseline characteristics after propensity score matching.

Characteristic	Romosozumab (*n* = 1201)	Denosumab (*n* = 1201)	*p* Value	SMD
Age at index, years	74.1 ± 9.1	74.2 ± 10.0	0.820	0.009
Female sex	1140 (94.9%)	1127 (93.8%)	0.249	0.047
White race	781 (65.0%)	769 (64.0%)	0.609	0.021
Asian race	155 (12.9%)	160 (13.3%)	0.762	0.012
Hypertensive disease	841 (70.0%)	820 (68.3%)	0.354	0.038
Ischemic heart disease	219 (18.2%)	223 (18.6%)	0.833	0.009
Atrial fibrillation/flutter	149 (12.4%)	149 (12.4%)	1.000	<0.001
Cerebrovascular disease	57 (4.7%)	51 (4.2%)	0.555	0.024
Heart failure	172 (14.3%)	175 (14.6%)	0.862	0.007
Diabetes mellitus	376 (31.3%)	366 (30.5%)	0.659	0.018
Serum creatinine, mg/dL	2.0 ± 9.5	1.4 ± 2.0	0.066	0.080
Hemoglobin A1c, %	6.1 ± 1.1	6.2 ± 1.2	0.016	0.141
HDL cholesterol, mg/dL	60.1 ± 20.3	56.9 ± 20.3	0.007	0.158
eGFR, mL/min/1.73 m^2^	52.6 ± 23.3	51.7 ± 24.3	0.388	0.037
Serum calcium, mg/dL	9.4 ± 0.6	9.5 ± 0.7	0.001	0.154

Data are presented as number (%) for categorical variables and mean ± SD for continuous variables. eGFR is presented as a continuous laboratory variable. eGFR, estimated glomerular filtration rate; HDL, high-density lipoprotein; SMD, standardized mean difference.

**Table 2:** Cardiovascular and survival outcomes through 1095 days after treatment initiation.

Outcome	Romosozumab Risk	Denosumab Risk	Risk Ratio (95% CI)	Hazard Ratio (95% CI)
Composite CV outcome	151/1201 (12.6%)	226/1201 (18.8%)	0.668 (0.553–0.808)	1.181 (0.958–1.455)
Cerebrovascular event	60/1201 (5.0%)	84/1201 (7.0%)	0.714 (0.518–0.985)	1.241 (0.886–1.738)
All-cause mortality	79/1201 (6.6%)	114/1201 (9.5%)	0.693 (0.526–0.913)	1.306 (0.976–1.747)
Acute myocardial infarction	46/1201 (3.8%)	75/1201 (6.2%)	0.613 (0.429–0.878)	1.096 (0.755–1.590)
Acute heart failure	32/1201 (2.7%)	73/1201 (6.1%)	0.438 (0.292–0.659)	0.720 (0.472–1.097)

The composite cardiovascular outcome included all-cause mortality, acute myocardial infarction, or cerebrovascular event. Hazard ratios are reported as complementary time-to-event estimates from TriNetX analyses; all hazard-ratio confidence intervals crossing 1.0. CV indicate cardiovascular.

## Data Availability

Restrictions apply to the availability of these data. Data were obtained from TriNetX Network and are available at https://trinetx.com/ the permission of TriNetX Network.

## Supplementary Materials

The following supporting information can be downloaded at: https://www.mdpi.com/article/10.3390/medicina62071302/s1. Table S1: Baseline characteristics after propensity score matching.

## Abbreviations

AMI: acute myocardial infarction; ARCH: Active-Controlled Fracture Study in Postmenopausal Women with Osteoporosis at High Risk; BMI: body mass index; CI: confidence interval; CKD: chronic kidney disease; CKD-MBD: chronic kidney disease-mineral and bone disorder; CV: cardiovascular; CVA: cerebrovascular accident/event; eGFR: estimated glomerular filtration rate; FRAME: Fracture Study in Postmenopausal Women with Osteoporosis; HF: heart failure; HR: hazard ratio; ICD-10-CM: International Classification of Diseases, Tenth Revision, Clinical Modification; MACE: major adverse cardiovascular event; PSM: propensity score matching; RANKL: receptor activator of nuclear factor kappa-B ligand; RD: risk difference; RR: risk ratio; SD: standard deviation; SMD: standardized mean difference.
