# Supplementary material for: Cardiovascular Outcomes Associated with Romosozumab Versus Denosumab in Chronic Kidney Disease
Source: Medicina (Kaunas). 2026 Jul 6;62(7):1302. doi: 10.3390/medicina62071302 (PMC13413852; doi:10.3390/medicina62071302)
Supplement: Supplementary file 1 [file medicina-62-01302-s001.zip › medicina-4360083-supplementary.pdf]

Supplementary Table S1. Baseline characteristics after propensity score matching.

Characteristic	Romosozumab (n = 1201)	Denosumab (n = 1201)	p Value	SMD
Age at index, years	74.1 ± 9.1	74.2 ± 10.0	0.820	0.009
Female sex	1140 (94.9%)	1127 (93.8%)	0.249	0.047
White race	781 (65.0%)	769 (64.0%)	0.609	0.021
Asian race	155 (12.9%)	160 (13.3%)	0.762	0.012
Hypertensive disease	841 (70.0%)	820 (68.3%)	0.354	0.038
Ischemic heart disease	219 (18.2%)	223 (18.6%)	0.833	0.009
Atrial fibrillation/flutter	149 (12.4%)	149 (12.4%)	1.000	<0.001
Cerebrovascular disease	57 (4.7%)	51 (4.2%)	0.555	0.024
Heart failure	172 (14.3%)	175 (14.6%)	0.862	0.007
Diabetes mellitus	376 (31.3%)	366 (30.5%)	0.659	0.018
Diuretics	388 (32.3%)	354 (29.5%)	0.133	0.061
Beta blockers	441 (36.7%)	430 (35.8%)	0.641	0.019
Calcium channel blockers	369 (30.7%)	336 (28.0%)	0.139	0.060
ACE inhibitors	131 (10.9%)	138 (11.5%)	0.651	0.018
Anticoagulants	356 (29.6%)	359 (29.9%)	0.894	0.005
Platelet aggregation inhibitors	265 (22.1%)	275 (22.9%)	0.625	0.020
Atorvastatin	271 (22.6%)	286 (23.8%)	0.468	0.030
Body mass index, kg/m^2	26.0 ± 6.4	26.1 ± 6.4	0.771	0.014
Systolic blood pressure, mm Hg	129.7 ± 21.0	131.4 ± 19.7	0.057	0.086
Serum creatinine, mg/dL	2.0 ± 9.5	1.4 ± 2.0	0.066	0.080
Hemoglobin A1c, %	6.1 ± 1.1	6.2 ± 1.2	0.016	0.141
Hemoglobin, g/dL	12.0 ± 1.8	12.0 ± 1.8	0.606	0.023
LDL cholesterol, mg/dL	90.4 ± 33.3	89.0 ± 31.5	0.434	0.045
HDL cholesterol, mg/dL	60.1 ± 20.3	56.9 ± 20.3	0.007	0.158
eGFR, mL/min/1.73 m^2	52.6 ± 23.3	51.7 ± 24.3	0.388	0.037
Serum calcium, mg/dL	9.4 ± 0.6	9.5 ± 0.7	0.001	0.154

Data are presented as number (%) for categorical variables and mean ± SD for continuous variables. eGFR is presented as a continuous laboratory variable. ACE indicates angiotensin-converting enzyme; eGFR, estimated glomerular filtration rate; HDL, high-density lipoprotein; LDL, low-density lipoprotein; SMD, standardized mean difference.
